# Dairy Product Intake and Long-Term Risk for Frailty among French Elderly Community Dwellers

**DOI:** 10.3390/nu13072151

**Published:** 2021-06-23

**Authors:** Berna Rahi, Hermine Pellay, Virginie Chuy, Catherine Helmer, Cecilia Samieri, Catherine Féart

**Affiliations:** 1 Department of Natural Sciences, School of Arts and Sciences, Lebanese American University, Byblos 36, Lebanon; berna.rahi@gmail.com; 2 Institut National de la Santé et de la Recherche Médicale (INSERM), University Bordeaux, INSERM, BPH, U1219, F-33000 Bordeaux, France; hermine.pellay@u-bordeaux.fr (H.P.); virginie.chuy@u-bordeaux.fr (V.C.); catherine.helmer@u-bordeaux.fr (C.H.); cecilia.samieri@u-bordeaux.fr (C.S.); 3 CNIEL, Service Recherche Nutrition-Santé, F-75009 Paris, France; 4 Department of Dentistry and Oral Health, CHU Bordeaux, University Bordeaux, F-33000 Bordeaux, France

**Keywords:** dairy products, frailty, older adults, cohort study

## Abstract

Dairy products (DP) are part of a food group that may contribute to the prevention of physical frailty. We aimed to investigate DP exposure, including total DP, milk, fresh DP and cheese, and their cross-sectional and prospective associations with physical frailty in community-dwelling older adults. The cross-sectional analysis was carried out on 1490 participants from the Three-City Bordeaux cohort. The 10-year frailty risk was examined in 823 initially non-frail participants. A food frequency questionnaire was used to assess DP exposure. Physical frailty was defined as the presence of at least 3 out of 5 criteria of the frailty phenotype: weight loss, exhaustion, slowness, weakness, and low physical activity. Among others, diet quality and protein intake were considered as confounders. The baseline mean age of participants was 74.1 y and 61% were females. Frailty prevalence and incidence were 4.2% and 18.2%, respectively. No significant associations were observed between consumption of total DP or DP sub-types and frailty prevalence or incidence (OR = 1.40, 95%CI 0.65–3.01 and OR = 1.75, 95%CI 0.42–1.32, for a total DP consumption >4 times/d, respectively). Despite the absence of beneficial associations of higher DP consumption on frailty, older adults are encouraged to follow the national recommendations regarding DP.

## 1. Introduction

In recent years, the world has been experiencing a steady increase in the aging population. It is expected that by 2050, one in six people will be over the age of 65, including one in four in Europe and northern America [1]. This increased life expectancy is associated with a higher risk of morbidities. In fact, nearly a quarter (23%) of the overall global burden of death and illness is in people aged over 60, and much of this burden is attributable to long-term illnesses [2]. Advancing age is indeed accompanied by common geriatric syndromes, such as frailty [3]. Frailty is characterized by a depletion in the functional reserves of physiological systems, which limits the possibility to adapt to changes in the environment over time, leading to falls, hospitalization, disability, and death [4]. Nevertheless, frailty can be prevented, and diet appears to be a major determinant of its development [5,6]. Several studies have reported that particular macronutrients [7,8], food groups [9,10,11] and dietary patterns are associated with frailty [12,13,14,15,16,17]. Particularly, our group has previously reported the relevance of protein intake (>1 g/d being associated with a lower prevalence of frailty) [18], of fruit and vegetable intake (>5 servings/d being associated with a lower risk of frailty) [9], and of the Mediterranean diet (a higher adherence being associated with a lower frailty risk) [17]. In line with our findings, several other longitudinal studies have showed that a higher protein intake is protective against frailty [19,20,21].

Dietary sources of protein include dairy products (DP), which are also important sources of calcium and vitamin D. Interestingly, recent studies have showed that higher DP consumption was associated with better age-related health outcomes, and particularly lower risks of type 2 diabetes [22,23], cardiovascular diseases and mortality [24,25]. The type of DP (i.e., milk, fresh DP and cheese) appears to be key component of such associations. In fact, a meta-analysis on 938,415 participants and 93,518 mortality cases reported an absence of association between total dairy (high- or low-fat) and milk with the risk of death, while total fermented dairy (including sour milk products, yogurt or cheese; +20 g/day) were associated with a significant 2% reduced risk of all-cause mortality and cardiovascular diseases [26]. While two systematic reviews also observed that higher DP intakes were associated with higher appendicular muscle mass, improved balance-test scores, and an attenuation of the loss of muscle strength [27,28], the direct potential benefit of DP on frailty as a whole has scarcely been studied. To the best of our knowledge, a single prospective study implemented in the Spanish Seniors-ENRICA cohort [29] reported that consuming seven or more servings per week of low-fat milk was associated with a significantly lower risk of frailty compared with consuming less than one serving per week. The external validity of such results remains uncertain. Indeed, the SHARE database demonstrated significant heterogeneity in DP consumption across Europe, with higher levels in central and northern countries and in Spain, and the lowest prevalence of dairy intake in eastern European countries [30]. Of note, high cheese consumption is a hallmark of French dietary habits, and France is also characterized by low milk consumption. Finally, several socio-demographic, nutritional characteristics and lifestyle factors have been associated with the French DP consumption, with specificities according to each DP sub-type [31]. Altogether, it is conceivable that the featured consumption of DP sub-types among French older adults could be differentially associated with frailty.

Therefore, our objective was to assess the cross-sectional and prospective associations between total DP and DP sub-types (milk, fresh DP and cheese) consumption and the 10-year frailty risk among older adults of the Three-City (3C) Bordeaux cohort.

## 2. Methods

### 2.1. Study Overview

The 3C-study is a French population-based prospective study initiated in 1999–2000 to study the vascular risk factors of dementia [32]. Its protocol was approved by the Consultative Committee for the Protection of Persons participating in Biomedical Research at Kremlin-Bicêtre and all participants gave written informed consent. Participants were randomly sampled from electoral rolls from three French cities (Bordeaux, Dijon, and Montpellier). Eligible participants had to be 65 years and older at the time of recruitment and not institutionalized. Among the 9294 participants included at baseline, 2104 were from the Bordeaux center, which completed the initial data collection in 2001–2002 (wave 1). A comprehensive dietary survey of 1597 participants was also performed. This dietary survey served as the baseline for the present study, where DP frequency of consumption and frailty were assessed.

### 2.2. Assessment of Dairy Products

Dietary data were obtained from a semi-quantitative Food Frequency Questionnaire (FFQ) administered during face-to-face interviews by dietitians. This allowed the assessment of the daily frequency of consumption of 148 foods and beverages (with frequencies assessed in 11 classes, from “never or less than once a month” to “7 times per week”) during each of the six meals/snacks of the day, as previously detailed [33]. Data from the FFQ was validated against a 24-h dietary recall in an independent subsample of the 3C-study [34]. DP consumption was considered using the frequency of consumption of milk, fresh DP, and cheese. The milk consumption variable included the consumption of “milk”, “coffee with milk”, “tea with milk”, “chocolate”, “chicory”, and “natural milk or with cereal”. Consumption of “yogurt and cottage cheese” was classified as fresh DP while frequency of consumption of “cheese” was considered as the cheese category. As already described by Pellay et al. (2020), we considered the DPs’ frequency of consumption as four main exposures, including total DP, milk, fresh DP, and cheese [31]. For each DP component, three categories were created based on the quartile distribution of consumption (low frequency: first quartile; intermediate frequency: quartiles 2 and 3; high frequency: fourth quartile). This classification ensured the differentiation between the most infrequent and frequent consumers, as previously described [31].

### 2.3. Assessment of Frailty

At baseline and at the 10-year follow-up, frailty was defined following the Cardiovascular Health Study frailty index [4], the tool recommended by the International Conference of Frailty and Sarcopenia Research [35]. Nevertheless, minor modifications were made to adapt this tool to the available data in our cohort study, as already published [17,18]. Briefly, (1) weight loss was defined as self-reported unintentional loss of 3 kg or more or, if missing, as a body mass index (BMI) <21 kg/m^2^; (2) exhaustion was evaluated using the following statements from the Center for Epidemiologic Studies-Depression scale (CES-D): “I felt that everything I did was an effort” and “I could not get going”. Participants were considered frail for this criterion when they answered “a moderate amount of the time” or “most of the time” to either of these statements [36]; (3) walking speed was determined based on a 6-m walking test, adjusting for height and gender. Participants in the highest quintile were considered slow. When this information was missing, participants were considered frail for this criterion when they reported being unable to walk between 500 m and 1 km or to walk up and down a flight of stairs based on the Rosow–Breslau scale [37]. This proxy has been shown to be strongly associated with walking [38]; (4) weakness was identified in different ways at baseline and at the 10-year follow-up, depending on availability of data. At the 10-year follow-up, weakness was identified using the handgrip strength quartiles stratified by sex and BMI, as recommended [4]. At baseline, weakness was identified using the chair standing method, shown to be a good proxy for handgrip strength [39]; (5) physical activity was assessed in a face-to-face interview via an open-ended questionnaire. Low physical activity was defined as less than 1 h of sports activities or less than 3.5 h of leisure activities per week, as previously described [17,18].

Older adults with three or more criteria out of five were considered as frail, otherwise they were considered as non-frail. Prevalent frail participants at baseline were excluded for the prospective analyses.

The FRAIL scale was also used to define frailty in sensitivity analyses [40]. The FRAIL scale includes five self-reported components: Fatigue, Resistance, Ambulation, Illnesses and Loss of weight. Fatigue and weight loss were evaluated similarly to those of the frailty index. Resistance and Ambulation were evaluated using the Rosow–Breslau scale, as recommended. Resistance was assessed by asking participants if they could walk up and down a flight of stairs and Ambulation by asking if they could walk between 500 m and 1 km; “no” responses were each scored as 1 point. Lastly, Illnesses was scored 1 for respondents who reported 5 or more chronic conditions out of 13 including hypertension, diabetes, hypercholesterolemia, cardio- and cerebro-vascular diseases (myocardial infarction or cardiac and vascular surgery, or arteritis or stroke), Parkinson’s disease, cognitive decline and dyspnea. Cancer was considered when reports were available, i.e., at the 10-year follow-up. The FRAIL score ranged from 0–5, with those scoring three or more considered as frail and those scoring two or less as non-frail.

### 2.4. Assessment of Disability

Dependency in basic Activities of Daily Living (ADLs) was assessed using the five following items of the Katz scale: bathing, dressing, toileting, transferring from bed to chair, and eating [41]. An individual was considered dependent if they could not perform at least one activity without a given level of assistance, as defined in the original instrument. All identified dependent participants at baseline and at 10-year follow-up were excluded from the analyses because frailty is considered as risk factor for dependency [35].

### 2.5. Covariates

The covariates included age, sex, marital status, education, smoking status, polypharmacy (dichotomous variable with 6 medications/d as a cut-off), multimorbidity (dichotomous variable with 2 chronic diseases or more as a cut-off point), and global cognitive performances using the Mini-Mental State Examination (MMSE) [42] (0–30 points; higher scores indicate better cognitive status). A diet quality score was also computed. This score included seven components: pulses, raw fruits, raw vegetables, cooked fruits and vegetables, fish, alcohol and olive oil. Each component was dichotomized into meeting the current dietary recommendations versus not. The total score of 7 was also dichotomized into having a good diet quality (score > 3) versus not (score ≤ 3). Finally, total protein intake was evaluated from a single 24-h dietary recall that was administered at home in addition to the FFQ [43].

### 2.6. Statistical Analysis

Baseline demographic, clinical and dietary characteristics were compared between prevalent frail and non-frail (i.e., sample used in the cross-sectional analysis) and incident frail and non-frail (i.e., sample used in the longitudinal analysis) older adults using the student’s *t*-test or chi-square test, depending on the type of the variables.

Logistic regression models were used to estimate odds ratio (OR) and 95% confidence intervals (95% CI) for the association between consumption of total DP or DP sub-type (milk, fresh DP, or cheese) and frailty, both cross-sectionally and prospectively. For each DP exposure, intermediate frequency consumption (quartiles 2 and 3) and high frequency consumption (quartile 4) were compared to the reference category of low frequency consumption (quartile 1).

Model 1 was adjusted for age, sex, education and marital status. Model 2 was additionally adjusted for smoking status, multimorbidity, polypharmacy, diet quality score, total protein intake and global cognitive performances. Finally, two sets of sensitivity analyses were performed. First, we assessed frailty using the FRAIL scale and the same multivariate models were applied, except excluding multimorbidity as a covariate from model 2 as this variable is a component of the FRAIL scale. Second, we retained all ADL dependent individuals as we assumed that those who are ADL dependent might already be frail, both in the cross-sectional analysis and the prospective one. All statistical analyses were performed with the SAS Statistical package (Version 9.4 SAS Institute) and statistical significance was set at *p* < 0.05.

## 3. Results

### 3.1. Sample Characteristics

**Figure 1:**
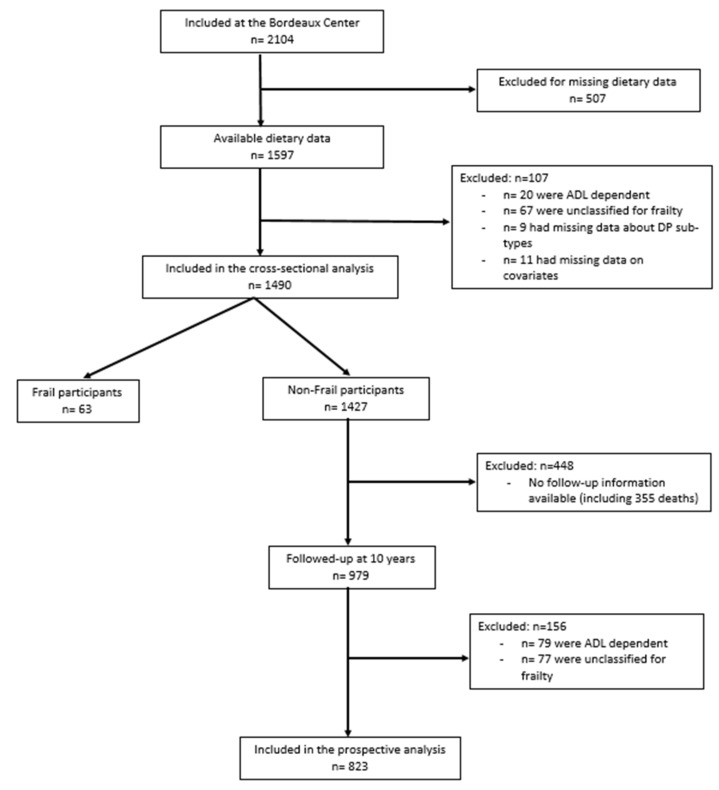
Flow chart of the cross-sectional and the prospective studied samples. Three-City Bordeaux Study, 2000–2010.

Among 1597 participants who answered the dietary survey at baseline, 107 were excluded from all analyses for the following reasons: 20 were ADL dependent at baseline, 67 could not be classified for frailty, 9 had missing information about DP consumption and 11 participants had missing information for covariates. Therefore, the final sample for the cross-sectional analysis comprised 1490 participants (including 1427 non-frail). Among those participants, 979 (69%) were followed up at 10 years (during the follow-up, 355 participants died). An additional 156 participants were excluded from longitudinal analyses (*n* = 79 participants were identified as dependent and *n* = 77 with missing frailty status at 10 years). Thus, 823 participants were prospectively analyzed (Figure 1).

In cross-sectional analyses, the studied sample (*n* = 1490) constituted mainly of females (*n* = 906, 60.8%) and had an average age of 74.1 ± 4.9 (standard deviation) years (Table 1). Half the sample was married (57%) and reported multimorbidity (48%), and a third (32%) was taking 6 medications/d or more. Prevalence of frailty was 4.2% (*n* = 63). The most prevalent frailty criterion was low physical activity (*n* = 234, 20.1%) followed by slow walking speed (*n* = 281, 19%) while the least prevalent frailty criterion was muscle weakness (*n* = 77, 5.3%) followed by weight loss (*n* = 82, 5.5%). Those included in prospective analyses (*n* = 823) were non-frail participants at baseline, mainly females (65.0%) and were on average 72.8 ± 4.4 years old. A total of 150 participants (18.2%) exhibited frailty at the 10-year follow-up and the most incident frailty criterion was low physical activity (*n* = 473, 58.2%) followed by muscle weakness (*n* = 199, 26.6%). The least incident frailty criterion was weight loss (*n* = 69, 8.4%) followed by exhaustion (*n* = 138, 17.6%).

Prevalent and incident frail older adults were significantly older, were more likely to be depressed, to take 6 medications/day or more, and to have comorbidities at baseline compared with prevalent non-frail participants, and with participants free from frailty over time, respectively (Table 1). Moreover, prevalent frail participants exhibited a significantly higher BMI on average than non-frail participants, and the daily consumption of proteins was not significantly different between the frail and non-frail participants at baseline (i.e., cross-sectional sample). Regarding the sample enrolled in prospective analyses, incident frail participants had a similar BMI compared to those who remained free from frailty, while a higher percentage of incident frail participants had a lower diet quality score compared with participants who remained free from frailty (53% vs. 44%, *p* = 0.045).

Frequencies of consumption of DP (total DP, milk, fresh DP, and cheese) are presented in Table 2 for both cross-sectional and prospective samples. No significant differences were observed between prevalent frail and non-frail or incident frail and non-frail participants regarding the frequency of total DP and DP-subtypes consumption at baseline.

### 3.2. Associations between Spectrum of DP Exposure and Prevalence of Frailty

In models adjusted for age, sex, marital status and education, we did not observe any significant association between total DP and DP sub-types and frailty prevalence, when comparing the highest frequency to the lowest frequency consumption of DP (Table 3). In models additionally adjusted for smoking status, multimorbidity, polypharmacy, protein intake, diet quality and global cognitive score, all associations with the prevalence of frailty remained not significant for all DP exposures: total DP (OR = 1.08, 95% CI = 0.54–2.17 and 1.40, 95% CI = 0.65–3.01 for intermediate and high consumption vs. low, respectively), milk (OR = 1.13, 95% CI = 0.56–2.31), fresh DP (OR = 1.13, 95% CI= 0.54–2.33), and cheese (OR = 0.89; 95% CI = 0.43–1.88) for high vs. low frequency of consumption.

### 3.3. Associations between Spectrum of DP Exposure and Incidence of Frailty

When focusing on the 10-year risk for frailty, we observed that baseline frequencies of consumption of total DP and DP sub-types were not significantly associated with the frailty risk when we compared the lowest frequency to the highest frequency of consumption of total DP (OR = 0.74, 95% CI = 0.42–1.30), milk (OR = 0.80, 95% CI = 0.48–1.35), fresh DP (OR = 0.68, 95% CI = 0.38–1.20) and cheese consumption (OR = 1.19, 05% CI = 0.68–2.10) in fully adjusted models (Table 4).

### 3.4. Sensitivity Analyses

The FRAIL scale was also implemented to alternatively identify prevalent and incident frail participants. Sixty out of 1552 participants (3.9%) were considered as frail at baseline according to this scale. In fully adjusted models (i.e., model 2), all associations with the prevalence of frailty were not significant for all DP exposures: total DP (OR = 1.42, 95% CI = 0.64–3.13), milk (OR= 1.11, 95% CI = 0.54–2.32), fresh DP (OR = 0.96, 95% CI= 0.46–1.98), and cheese (OR = 0.86; 95% CI = 0.39–1.88) for high vs. low frequency of consumption. Among 1492 non-frail non-dependent, 1006 were followed at the 10-year follow-up (lost to follow-up *n* = 486). Among those, an additional 87 were excluded from the analysis because they were ADL dependent and another 23 were excluded because they were unclassified for the FRAIL scale, leading to a final sample size of 896, with 45 (5.0%) classified as frail on the FRAIL scale. Regarding the spectrum of DP exposures, we only observed that the highest, compared with the lowest, frequency of consumption of fresh DP was associated with lower frailty risk in the fully adjusted model (OR = 0.35, 95% CI = 0.13–0.97, *p* = 0.04, *p* global = 0.13) while all other associations were non-significant, for instance, total DP (OR = 1.66, 95% CI = 0.26–1.67), milk (OR= 1.21, 95% CI = 0.49–2.98), and cheese (OR = 1.25; 95% CI = 0.49–3.21) for high vs. low frequency of consumption.

Second, when all ADL dependent individuals were maintained in analytic samples, 1501 and 885 participants were included for the cross-sectional and prospective analyses, respectively. Among those 1501 participants, 68 (4.5%) were identified as frail. None of the total DP or DP sub-types exposures were associated with frailty prevalence in the fully adjusted models: total DP (OR = 1.44, 95% CI = 0.68–3.04), milk (OR= 1.04, 95% CI = 0.52–2.1), fresh DP (OR = 1.21, 95% CI= 0.59–2.48), and cheese (OR = 0.85; 95% CI = 0.42–1.74) for high vs. low frequency of consumption. Among those 1433 non-frail at baseline, 449 were lost at the 10-year follow-up and 99 were unclassified for frailty incidence, leading to a final sample size of 885 for the prospective analyses. At the 10-year follow-up, 195 participants (22%) were identified as frail. Regarding the spectrum of DP exposures, none of the frequency of consumption of total DP or DP sub-types were significantly associated with frailty risk in the fully adjusted models: total DP (OR = 0.65, 95% CI = 0.39–1.09), milk (OR = 0.61, 95% CI = 0.38–1.00), fresh DP (OR = 0.64, 95% CI = 0.39–1.08), and cheese (OR = 1.4; 95% CI = 0.83–2.41) for high vs. low frequency of consumption.

## 4. Discussion

In the present analysis of French community-dwelling older adults enrolled in the 3C-Bordeaux study, the frequency of DP consumption was not significantly associated with frailty, assessed using proxies of the frailty phenotype, in either cross-sectional or prospective analyses. In particular, total DP, milk, fresh DP and cheese were not associated with frailty prevalence at baseline. Similarly, these food groups were not associated with frailty risk at 10 years. Similar results were observed when frailty was assessed using the FRAIL scale, strengthening our conclusions.

Several studies have evaluated the association between DP and age-related chronic diseases and mortality [22,23,44]. Nevertheless, to our knowledge, very few studies have evaluated the relationship between DP and frailty and their results were mixed. A cross-sectional study evaluated the association between dairy intake and physical function among1456 older women aged 70 to 85 years [45]. The authors observed that compared to those in the lowest tertile of dairy consumption, those in the highest tertile of consumption had significantly higher handgrip strength and lower odds for a poor Timed Up and Go while no differences were observed in the prevalence of falls. In a sample of 1871 Spanish older adults enrolled in the Seniors-ENRICA cohort [29], greater consumption of low-fat dairy products and low-fat milk in particular was associated with lower frailty risk over 3.5 years of follow-up, while no significant results were observed for whole milk, yogurt, cheese and low-fat yogurt. Contrary to the presented studies, our findings did not show any association with frailty prevalence or risk at the 10-year follow-up. Interestingly, our results are similar to a recent analysis of the InCHIANTI study where the main objective was to evaluate the associations between adherence to a Mediterranean-type diet (MeDi) and frailty index at baseline and at the 10-year follow-up [46]. In a sub-analysis, the authors investigated the effect of individual components of the MeDi and frailty, and they observed that DP intake was not significantly associated with frailty in both the cross-sectional and prospective analyses.

Several possible explanations could justify the absence of associations between DP and frailty in our sample. First, the FFQ used to collect dietary data assessed the frequency of consumption only, while information about the quantities, which could have been interesting, were only assessed by a single 24-h dietary recall (which questions its relevance). Therefore, despite the higher consumption frequency, this intake might have been below what is recommended and therefore affected our results. In fact, in a recent analysis of the 3C-study participants describing their DP intake at baseline, it was observed that participants with the highest frequency of total DP per day consumed lower than the recommended intakes [31]. These results were in line with a previous national report where it was observed that 64% of participants aged 55 to 79 years old reported consumption below recommendations [47]. Second, in the 3C-study, total DP consumption and its sub-types have been previously shown to be associated with different eating patterns [31]. Although we have adjusted for diet quality in our analyses, we cannot exclude the possibility of some residual confounding that has led to an absence of significance. This is noteworthy as it was observed that higher total DP consumption was associated with a higher consumption of biscuits, sweets and cooked vegetables, and higher frequency of milk consumption was significantly associated with higher intakes of biscuit and sweets, a dietary pattern described as “biscuits and snacking” in the 3C-study. Moreover, it was observed that the highest frequency consumers of fresh DP had a low total energy intake. Finally, the highest frequency of cheese consumption was associated with a high consumption of cereals and grains, sweets, charcuterie, meat, poultry, and alcohol [31]. These results showed that the higher consumption of total DP or sub-types was associated with less-than-optimal diets, rich in sugar and saturated fatty acids, part of a western-type diet [48] and potential risk factors for frailty [14,49,50]. For instance, in a cross-sectional NHANES study including 4062 participants ≥50 y of age, a higher percentage of saturated fatty acid intake was associated with higher frailtyprevalence [50]. Therefore, we speculated that the null association between highest DP consumption and frailty observed here might be the result of possible positive effects of some favorable nutrients on frailty (i.e., higher protein and energy intake), but attenuated by the possible negative effects of saturated fatty acid intake and the overall diet quality, although we have controlled for components of the diet in the analyses. Third, no information was available about the quality of DP, whether they were natural or sweetened, or fermented or not. In fact, flavored milk, whole yogurt and fermented milk, dairy desserts and sweetened cheeses are all sources of added sugars, which were shown to be associated with an increased risk of frailty in an analysis of the Seniors ENRICA cohort [49]. The highest tertile of added sugars consumption was associated with a higher frailty risk (i.e., multiplied by 2.3) compared to those in the lowest tertile. Finally, unlike the analyses from the Seniors-ENRICA cohort study [29], we were not able to differentiate between types of DP consumed based on their fat content. Nevertheless, the 24-h dietary recall administered at baseline of the 3C-Bordeaux study showed that only 7% of the participants had whole-fat milk and among those, only 10% reported regular consumption of whole-fat milk while up to 25% of the sample consumed whole fresh DP and 19% consumed flavored fresh DP or yogurt with fruits (unpublished data). This might imply that factors other than the fat content of DP might play a role in the association between DP and frailty. Altogether, we speculated that the observed null association between highest DP consumption (whatever the subtypes) and frailty might be the results of interactions between the different concentrations of beneficial and harmful ingredients leading to an unbalanced quality of DP and of related dietary patterns.

The present study has some methodological limitations. First, as previously stated, we had no detailed information about the portion sizes and this would have affected our results as national recommendations emphasize the quantity consumed rather than frequency. Moreover, a high frequency consumption does not necessarily mean reaching the recommended levels, as older adults might have frequent but smaller intakes. Therefore, the inability to evaluate the portion sizes might have hindered any potential association of DP with frailty status. We also did not assess DP intake from mixed dishes, and this might have led to underestimation of the DP consumption frequency. This is an important issue to consider in future studies as milk is a recurrent constituent of several French recipes. Another limitation is that we did not adjust for important micronutrients related to DP and associated with frailty, namely, vitamin D [51,52]. Furthermore, recall bias cannot be excluded as it can lead to under or overestimation of DP intakes despite meticulous data collection. Regarding the assessment of frailty, we complemented slowness and handgrip strength with the Rosow–Breslau scale and the chair stand test, respectively, to minimize the loss of participants due to missing data. Indeed, the Rosow–Breslau scale has been shown to be strongly associated with walking [38] and the chair stand test was shown to be a good proxy for handgrip strength [39,53]. Furthermore, we were not able to check frailty incidence over 10-years at different waves of follow-up because the frailty phenotype could not be calculated at each time interval. Nevertheless, this limit was toned down when using the FRAIL scale, which identified a lower number of frail participants, but provided similar results on the DP-frailty associations in both the cross-sectional and prospective analyses. Despite these similarities, we speculate that the imbalance between frail and non-frail groups might have led to underpowered comparisons, hindering the observation of real differences if any. In addition, a selection bias cannot be dismissed, since not included participants (cross-sectional sample) were older, had lower educational levels and cognitive performance, had more frequent depressive symptoms, multi-morbidities, polypharmacy, and worse diet scores than included participants (data not shown). Finally, although we adjusted for several major covariates, some residual confounding factors cannot be dismissed. In fact, we acknowledge that the collected dietary data dates back to 2000, which is old, and this might affect the relevance of our results. Nevertheless, the French RDA applied to the year of data collection (2000–2001) is still applied till now and it has been previously reported that intakes of major food groups appeared to be relatively stable during follow-up in 3C Bordeaux [54].

Despite these limitations, the current study has several strengths. First, we focused our analyses on a large sample of French elderly consumers, known to exhibit distinctive DP consumption, notably cheese [31], within a population-based setting while adjusting for major confounders (note, less than 0.1% of 3C-Bordeaux participants were consumers of food supplements at baseline, which precluded using this data as a confounder). Second, survival analyses were performed to check if there is any competitive risk with death (data not shown). We observed that DP exposures were not significantly associated with mortality, eliminating the selection bias leading to survival effect often faced in prospective studies involving older adults. Moreover, we confirmed our main results using a different scale to assess frailty and when keeping participants who exhibited dependency in both cross-sectional and prospective studied samples, which allowed us to further decrease the selection bias (i.e., frailty being considered as a pre-dependency stage and risk factor for disability [35,55]).

In conclusion, we did not observe any association between DP consumption, whatever the sub-types, and frailty prevalence or incidence among this sample of French older adults. Studies on this topic are scarce and future studies are still needed while taking into consideration the identified limitations, such as the potential benefits/risks ratio of DP nutrient contents. In the meantime, and beyond frailty, older adults are encouraged to follow French nutritional recommendations for DP consumption (2 to 3 times/d) as their benefits on the general well-being of older adults to prevent osteoporosis and malnutrition are well established, and recent large-scale settings have also suggested their protective ability to prevent chronic diseases and mortality.

## Figures and Tables

**Table 1:** Socio-demographic, clinical, dietary, and lifestyle characteristics according to the frailty prevalence (cross-sectional sample, *n* = 1490 in 2000) and incidence (prospective sample, *n* = 823 between 2000–2010) of older adults from the Three-City study, Bordeaux (France).

	Cross-Sectional Analyses				Prospective Analyses			
	Overall (*n* = 1490)	Frail at Baseline (*n* = 63)	Non-Frail at Baseline (*n* = 1427)	*p* ^a^	Overall (*n* = 823)	Non-Frail at Follow-Up (*n* = 673)	Incident Frail (*n* = 150)	*p* ^a^
Demographic characteristics (*n*, %) *								
Sex (females)	906 (60.81)	43 (68.25)	863 (60.48)	0.22	535 (65.01)	433 (64.34)	102 (68.00)	0.40
Age (y), mean (SD)	74.10 (4.90)	77.69 (5.24)	73.95 (4.82)	<0.0001	72.80 (4.35)	72.20 (4.08)	75.49 (4.50)	<0.0001
Education				0.27				0.11
No/Primary	478 (32.10)	26 (41.27)	452 (31.67)		232 (28.19)	180 (26.75)	52 (34.67)	
Secondary or high	731 (49.10)	26 (41.27)	705 (49.40)		424 (51.52)	357 (53.05)	67 (44.67)	
University	281 (18.90)	11 (17.46)	270 (18.92)		167 (20.29)	136 (20.21)	31 (20.67)	
Marital Status				0.053				0.001
Married	849 (57.00)	26 (41.27)	823 (57.67)		471(57.23)	402 (59.73)	69 (46.00)	
Divorced/separated	118 (7.90)	5 (7.94)	113 (7.92)		64 (7.78)	56 (8.32)	8 (5.33)	
Widowed	429 (28.80)	27 (42.86)	402 (28.17)		228 (27.70)	168 (24.96)	60 (40.00)	
Single	94 (6.30)	5 (7.94)	89 (6.24)		60 (7.29)	47 (6.98)	13 (8.67)	
Clinical characteristics (*n*, %) *								
Smoking status				0.45				0.54
Never smoker	937 (62.90)	44 (69.84)	893 (62.58)		540 (65.61)	442 (65.68)	98 (65.33)	
Ex-smoker	472 (31.70)	17 (26.98)	455 (31.89)		242 (29.40)	195 (28.97)	47 (31.33)	
Current	81 (5.40)	2 (3.17)	79 (5.54)		41 (4.98)	36 (5.35)	5 (3.33)	
MMSE score ^b^, mean (SD)	27.52 (1.98)	26.43 (2.34)	27.57 (1.96)	<0.0001	27.84 (1.79)	27.89 (1.76)	27.61 (1.90)	0.08
Medications/d ≥ 6	480 (32.20)	38 (60.31)	442 (30.97)	<0.0001	220 (26.73)	155 (23.03)	65 (43.33)	<0.0001
Multimorbidity	727 (48.8)	41 (65.08)	686 (48.07)	0.008	395 (48.00)	299 (44.43)	96 (64.00)	<0.0001
Nutritional and dietary characteristics								
BMI (kg/m^2^) (m = 18)	26.33 (4.16)	27.51 (6.10)	26.28 (4.06)	0.03	26.25 (3.95)	26.15 (3.83)	26.68 (4.43)	0.15
BMI categories				0.16				0.63
<23	307 (20.86)	12 (21.05)	295 (20.85)		172 (21.00)	142 (21.19)	30 (20.13)	
23–27	577 (39.20)	16 (28.07)	561 (39.65)		329(40.17)	273 (40.75)	56 (37.58)	
>27	588 (39.95)	29 (50.88)	559 (39.51)		318 (38.83)	255 (38.06)	63 (42.28)	
Total protein (g/d)	75.75 (26.80)	72.14 (27.05)	75.91 (26.79)	0.27	75.36 (26.22)	76.05 (25.98)	72.23 (27.12)	0.11
Diet index (*n*, % high quality)	761 (51.1)	32 (50.79)	729 (51.09)	0.96	423 (51.40)	357 (53.05)	66 (44.00)	0.05
Items of the phenotype of frailty								
Weight loss	82 (5.51) (m = 1)	27 (43.55)	55 (3.85)	<0.0001	69 (8.42) m = 4	35 (5.22)	34 (22.97)	<0.0001
Exhaustion	223 (15.12) (m = 15)	53 (84.13)	170 (12.04)	<0.0001	138 (17.56) m = 37	54 (8.32)	84 (61.31)	<0.0001
Muscle weakness	77 (5.25) (m = 24)	35 (58.33)	42 (2.99)	<0.0001	199 (26.57) m = 74	107 (17.31)	92 (70.23)	<0.0001
Walking speed	281 (18.90) (m = 3)	53 (84.12)	228 (16.01)	<0.0001	209 (25.49) m = 3	81 (12.09)	128 (85.33)	<0.0001
Physical activity	234 (20.05) (m = 323)	36 (78.26)	198 (17.66)	<0.0001	473 (58.18) m = 10	329 (49.47)	144 (97.30)	<0.0001

* All data are presented as *n* (%) except for age, MMSE, BMI, and protein intake where the mean (SD) is presented; ^a^ Baseline differences between prevalent frail and non-frail (*n* = 1490) and incident frail and non-frail (*n* = 823) participants tested by *t*-tests or chi square tests depending on the type of the variable; ^b^ Mini-Mental State Examination; m = missing.

**Table 2:** Frequency of consumption of dairy products (total and sub-types) according to the frailty prevalence (cross-sectional sample, *n* = 1490 in 2000) and incidence (prospective sample, *n* = 823 between 2000–2010) of older adults from the Three-City study, Bordeaux (France).

	Cross-Sectional Analyses				Prospective Analyses			
	Overall (*n* = 1490)	Frail at Baseline (*n* = 63)	Non-Frail at Baseline (*n* = 1427)	*p* ^a^	Overall (*n* = 823)	Non-Frail at Follow-Up (*n* = 673)	Incident Frail (*n* = 150)	*p* ^a^
Total Dairy Products				0.37				0.58
Low: ≤2 times/d	375 (25.17)	13 (20.63)	362 (25.37)		198 (24.06)	157 (23.33)	41 (27.33)	
Intermediate: 2–4 times/d	769 (51.61)	31 (49.21)	738 (51.72)		439 (53.34)	362 (53.79)	77 (51.33)	
High: ≥4 times/d	346 (23.22)	19 (30.16)	327 (22.92)		186 (22.60)	154 (22.88)	32 (21.33)	
Milk				0.37				0.54
Low: 0 times/d	433 (29.06)	17 (26.98)	416 (29.15)		229 (27.83)	182 (27.04)	47 (31.33)	
Intermediate: 0–1 times/d	716 (48.05)	27 (42.86)	689 (48.28)		383 (46.54)	318 (47.25)	65 (43.33)	
High: >1 times/d	341 (22.89)	19 (30.16)	322 (22.56)		211 (25.64)	173 (25.71)	38 (25.33)	
Fresh dairy products				0.52				0.46
Low: <0.5 times/d	408 (27.38)	16 (25.40)	392 (27.47)		207 (25.15)	167 (24.81)	40 (26.67)	
Intermediate: 0.5–1.5 times/d	723 (48.52)	28 (44.44)	695 (48.70)		419 (50.91)	339 (50.37)	80 (53.33)	
High: >1.5 times/d	359 (24.09)	19 (30.16)	340 (23.83)		197 (23.94)	167 (24.81)	30 (20.00)	
Cheese				0.39				0.31
Low: ≤0.5 times/d	297 (19.93)	16 (19.69)	281 (19.69)		174 (21.14)	143 (21.25)	31 (20.67)	
Intermediate: 0.5–1.5 times/d	779 (52.28)	28 (52.63)	751 (52.63)		477 (54.31)	372 (55.27)	75 (50.00)	
High: >1.5 times/d	414 (27.79)	19 (30.16)	395 (27.68)		202 (24.54)	158 (23.48)	44 (29.33)	

All data are presented as *n* (%); ^a^ Baseline differences between prevalent frail and non-frail (*n* = 1490) and incident frail and non-frail (*n* = 823) participants tested by *t*-tests or chi square tests depending on the type of the variable; m = missing.

**Table 3:** Multivariate association between baseline frequencies of consumption of total DP, milk, fresh DP, and cheese and frailty prevalence among older adults in the Three-City Study, Bordeaux (*n* = 1490, 2000).

**Total Dairy Products Frequency of Consumption**					
	*n* frail/Total	Model 1		Model 2	
		OR (95% CI)	*p*	OR (95% CI)	*p*
Low: ≤2 times/d	13/375	Ref		Ref	
Intermediate: 2–4 times/d	31/769	1.07 (0.54–2.09)	0.84	1.08 (0.54–2.17)	0.82
High: ≥4 times/d	19/346	1.50 (0.72–3.13)	0.28	1.40 (0.65–3.01)	0.39
Global p			0.45		0.62
**Milk Frequency of Consumption**					
	*n* frail/Total	Model 1		Model 2	
		OR (95% CI)	*p*	OR (95% CI)	*p*
Low: 0 times/d	17/433	Ref		Ref	
Intermediate: 0–1 times/d	27/716	0.91 (0.49–1.71)	0.77	0.83 (0.44–1.58)	0.57
High: >1 times/d	19/341	1.21 (0.61–2.41)	0.58	1.13 (0.56–2.31)	0.73
Global p			0.66		0.62
**Fresh Dairy Products Frequency of Consumption**					
	*n* frail/Total	Model 1		Model 2	
		OR (95% CI)	*p*	OR (95% CI)	*p*
Low: <0.5 times/d	16/408	Ref		Ref	
Intermediate: 0.5–1.5 times/d	28/723	0.90 (0.47–1.72)	0.76	0.83 (0.43–1.62)	0.57
High: >1.5 times/d	19/359	1.22 (0.60–2.48)	0.58	1.13 (0.54–2.33)	0.74
Global p			0.62		0.62
**Cheese Frequency of Consumption**					
	*n* frail/Total	Model 1		Model 2	
		OR (95% CI)	*p*	OR (95% CI)	*p*
Low: ≤0.5 times/d	16/297	Ref		Ref	
Intermediate: 0.5–1.5 times/d	28/779	0.66 (0.35–1.27)	0.22	0.68 (0.38–1.32)	0.25
High: >1.5 times/d	19/414	0.92 (0.45–1.86)	0.81	0.89 (0.43–1.88)	0.77
Global p			0.38		0.45

OR: Odds ratio, CI: Confidence Intervals; Model 1: Model adjusted for age, sex, marital status and education; Model 2: Model 1 + additional adjustment for smoking status, multimorbidity, polypharmacy, total protein, diet quality score, and Mini-Mental State Examination.

**Table 4:** Multivariate association between baseline frequencies of consumption of total DP, milk, fresh DP, and cheese and the 10-year frailty risk among older adults in the Three-City Study, Bordeaux (*n* = 823, 2000–2010).

**Total Dairy Products Frequency of Consumption**					
	*n* frail/Total	Model 1		Model 2	
		OR (95% CI)	*p*	OR (95% CI)	*p*
Low: ≤2 times/d	41/198	Ref		Ref	
Intermediate: 2–4 times/d	77/439	0.70 (0.44–1.09)	0.11	0.73 (0.46–1.16)	0.19
High: ≥ 4 times/d	32/186	0.76 (0.44–1.30)	0.19	0.75 (0.42–1.32)	0.32
Global p			0.28		0.40
**Milk Frequency of Consumption**					
	*n* frail/Total	Model 1		Model 2	
		OR (95% CI)	*p*	OR (95% CI)	*p*
Low: 0 times/d	47/229	Ref		Ref	
Intermediate: 0–1 times/d	65/383	0.47 (0.50–1.21)	0.26	0.79 (0.50–1.22)	0.28
High: >1 times/d	38/211	0.75 (0.45–1.24)	0.26	0.78 (0.46–1.31)	0.34
Global p			0.43		0.50
**Fresh Dairy Products Frequency of Consumption**					
	*n* frail/Total	Model 1		Model 2	
		OR (95% CI)	*p*	OR (95% CI)	*p*
Low: <0.5 times/d	40/207	Ref		Ref	
Intermediate: 0.5–1.5 times/d	80/419	0.92 (0.58–1.44)	0.71	0.91 (0.57–1.45)	0.68
High: >1.5 times/d	30/197	0.66 (0.38–1.16)	0.15	0.65 (0.57–1.16)	0.15
Global p			0.31		0.31
**Cheese Frequency of Consumption**					
	*n* frail/Total	Model 1		Model 2	
		OR (95% CI)	*p*	OR (95% CI)	*p*
Low: ≤0.5 times/d	31/174	Ref		Ref	
Intermediate: 0.5–1.5 times/d	75/447	0.86 (0.53–1.40)	0.54	0.86 (0.52–1.41)	0.54
High: >1.5 times/d	44/202	1.31 (0.76–2.27)	0.33	1.25 (0.71–2.21)	0.44
Global p			0.17		0.27

OR: Odds ratio, CI: Confidence Intervals; Model 1: Model adjusted for age, sex, marital status and education; Model 2: Model 1 + additional adjustment for smoking status, multimorbidity, polypharmacy, total protein, diet quality score, and Mini-Mental State Examination.

## Data Availability

Data described in the manuscript, code book and analytic code will be made available upon request: http://www.three-city-study.com/ancillary-studies.php (accessed on 5 April 2021).
